# Examining China’s public health discourse on juvenile myopia issues: a multimodal critical discourse analysis on a public health promotion advertisement

**DOI:** 10.3389/fpubh.2025.1586978

**Published:** 2025-09-04

**Authors:** Tianxiong Lyu

**Affiliations:** Institute of Language Sciences, Shanghai International Studies University, Shanghai, China

**Keywords:** myopia, China, multimodal critical discourse analysis, neoliberalism, biopedagogy

## Abstract

This article analyses a public health promotion advertisement on juvenile myopia issues, which stands out as one of the most widely viewed among those addressing juvenile myopia prevention released by the National Health Commission of the People’s Republic of China (NHC) on bilibili, a prominent video-sharing platform in China. The article discusses how a designed public health discourse on juvenile myopia issues is constructed through various techniques and what ideologies can be reflected through this discourse. Employing a multimodal critical discourse analysis (MCDA) approach based on the three-dimensional discourse framework, this article underscores how the advertisement intricately employs diverse semiotic resources to craft its discursive script. Notably, through designed narrative genres, characters, scenes, and settings, the ad strategically amplifies the role of adult parents as pivotal agents in preventing juvenile myopia, thus legitimizing their primary responsibility. However, this emphasis on parental responsibility subtly obscures the influence exerted by other societal actors and social factors in the myopia prevention landscape. Through this nuanced analysis, the study sheds light on the attempt of public policy discourse hidden in the advertisement to shape perceptions and attitudes toward myopia prevention, underscoring its underlying intention to advance the tenets of neoliberal biopedagogy.

## 1 Introduction

In the field of public health policy and public health communication, scholars with a critical perspective are increasingly concerned with the emphasis on individual empowerment and responsibility in public health, as well as the neglect of action and structural reform, and the so-called “healthy lifestyles” (1), which highlights personal issues, paternalism, and victim-blaming tendencies (2, 3). Numerous studies related to this in public health discourse in Western countries reveal that the responsibility for public health has shifted, creating a culture of blame-shifting.

This transformation in public-health-related discourse rooted in neoliberalism and the consequent shift in citizenship is evident not only in Western countries but also in China. Against China’s gradual privatization and stabilization (4), inculcating personal health responsibility, disseminating health knowledge, and encouraging health practices have become common themes in Chinese mass commercial media (5). Relevant Chinese government departments actively participate in information dissemination, using media to spread the public health knowledge they advocate. However, compared to the extensive discussions of related discourses in Western social contexts based on Western public health issues, the exploration of relevant discourses concerning Chinese public health issues remains relatively insufficient in the Chinese context.

Among the many public health problems facing Chinese society today, the juvenile myopia issue has been identified by the Chinese government (6) as “a significant issue affecting the future of the nation and its people.” Based on the statistical data from the NHC, in 2020, the overall myopia rate among children and adolescents in China was 52.7%, showing a 2.5% increase compared to 2019. Specifically, the myopia rates have reached 14.3% for 6-year-old children, 35.6% for primary school students, 71.1% for junior high school students, and 80.5% for high school students (7). These data all indicate that China is becoming one of the countries with the most severe myopia problems in the world.

To address this problem, the National Health Commission of the People’s Republic of China (NHC) has successively released public health promotion advertisements on myopia prevention on various online social platforms, including Weibo, WeChat Video, bilibili, and others, to disseminate and promote knowledge about myopia prevention and treatment from multiple perspectives. These advertisements, as a recontextualization of the government’s public health policy using multimodal resources, not only reflect the intentions of the power but also imply the ideologies in the public health communication process. Analyzing these advertisements helps to reveal the embedded discursive strategies, elucidating how these discourses reframe the issue of adolescent myopia and the intentions behind such reframing.

As a result, this paper will analyze the publicity campaign on juvenile myopia issues, with a specific focus on public health promotion advertisements produced and promoted by the NHC. To be specific, this paper will discuss the following two questions: (a) What kind of public health discourse is constructed through this advertisement, and (b) What ideologies or intentions are reflected through the advertisement?

## 2 Myopia in China

Myopia, also known as near-sightedness and short-sightedness, is an eye disease resulting from the elongation of the eyeball or the lens becoming too strong. According to the World Health Organization (WHO)'s World Report on Vision (8), the prevalence of myopia in East Asia has reached 51.6%, making it one of the regions with the highest myopia rates worldwide. As the most populous country in the region, China faces a severe issue of myopia, where approximately 80% of students who finish 12 years of schooling currently experience near-sightedness (9).

While individual and genetic factors can influence myopia development, postnatal behaviors, environmental factors, and social elements also play a role in this process. Activities involving close-range work, such as reading, writing, and computer use, are significant contributors to myopia’s increased prevalence (10). Additionally, enclosed housing in urban areas (11) and the rapid increase in the use of computers and mobile phones (12) may also be associated with myopia development. Some studies indicate that factors such as family income, parental occupation, parental education level, and community environment are correlated with myopia and other vision loss issues (13–15). Moreover, considering the relatively high myopia rates in East Asia countries, including China, many scholars associate this with the high-pressure school systems and local parents’ special emphasis on academic performance in this region (9, 16, 17). These indicate that myopia is not only an individual health issue but also a public concern involving various factors, such as the education system, economic status, social environment, and local culture.

Believing that the persistently high myopia rate among children and adolescents has become “a significant issue affecting the future of the nation and its people,” the Chinese government (6) has issued the Comprehensive Implementation Plan for the Prevention and Control of Myopia in Children and Adolescents, requiring various relevant ministries and stakeholders to cooperate with each other to safeguard the visual health of minors. The policy document requires actions from families, schools, medical institutions, juvenile individuals, and relevant departments to address the public health issue of myopia in minors. Similarly, in other related policy documents subsequently issued, like The Action Plan for the Prevention and Control of Myopia in Children and Adolescents 2021–2025 (18), it is also emphasized that the issue of myopia in minors involves various sectors of society and requires collective efforts to address it. One of the requirements mentioned in these policy documents is to utilize forms like public service advertisements to disseminate and promote knowledge about myopia prevention and treatment from multiple perspectives. To meet this requirement, the NHC has successively released public health promotion advertisements on myopia prevention on various online social platforms, including Weibo, WeChat Video, bilibili, and others. These advertisements can be identified as typical examples reflecting the Chinese government’s public health discourse on juvenile myopia issues and the ideologies hidden behind it.

## 3 Neoliberalism and biopedagogy

Although the forms and patterns vary in different countries, it is undeniable that neoliberalism is rapidly spreading as a powerful force, influencing the ways in which economies and societies operate, and reshaping the concepts of government and citizenship (19). As a political rationality that emerged in response to the economic crises of the 1970s, neoliberalism is based on a particular market concept, which is now continuously influencing various domains. However, its governance and social organization are not merely the result of its penetration from the economic sphere into other areas. Instead, it is the outcome of explicitly imposing a particular form of market rationality on other domains (20). One of the corresponding manifestations is the emphasis on individual responsibility.

Policies associated with neoliberalism usually produce citizens as individual consumers or entrepreneurs whose moral autonomy is determined by their ability to “self-care,” which means to meet their own needs and pursue their own goals (20). Under this ideology, individuals become the primary agents responsible for their own health, development, success, and happiness (21), and the prioritization of collective and societal responsibilities and interests is significantly diminished. However, this does not imply that the political objectives of neoliberalism aim to achieve a small government. Under the guise of advocating for individual rights and responsibilities, neoliberalism is, in fact, gradually transferring public wealth into the hands of a small elite, while simultaneously shifting the political, economic, and social responsibilities of the government onto individuals (22).

With the proliferation of neoliberalism, this ideology has also impacted public policy and the field of public health, reshaping discourse within the health communication process. Intentionally or unintentionally, it tends to make individuals or groups perceived as inherently responsible for addressing certain societal issues. This phenomenon is reflected in research on public health policies and discourses related to epidemics such as obesity (23–25) and COVID-19 (26, 27).

A repeated discussion of a crucial factor in the context of related issues is biopedagogy. Foucault (28, 29) discusses the concept of “biopower,” emphasizing that citizens are encouraged to internalize knowledge deemed “normal” regarding sexuality, criminality, reproduction, and more, leading to the subsequent monitoring and control of their behavior. Based on this, Harwood (30) introduces the concept of “biopedagogy.” This concept emphasizes the use of empirical analysis to uncover and question the biopolitical practices hidden behind governance discourse, which employ symbolic expressions of health knowledge in normative, socially regulatory, and reproductive ways, thereby recontextualizing health knowledge (25). Through this way, the idea of “healthism,” that personal health should and can be earned by individual efforts (31), can be sowed in people’s minds, and voluntary care for the body and prevention of policy opposition can be achieved (23).

## 4 Data and methods

Since biopedagogy often manifests in a concealed manner within discourse, revealing the underlying intentions and values of the related discourse may require the application of critical discourse analysis (CDA). Fairclough (32) proposes a three-dimensional discourse framework for discourse analysis, involving analysis across three dimensions: text, discursive practice, and social practice, which can serve as a powerful model for revealing the relationship between discourse text and power and ideology. Considering that the data involved in this study is a public health promotion advertisement video encompassing various semiotic resources such as textual, audio, and visual elements, the research has opted for a multimodal critical discourse analysis (MCDA) approach (19, 33), which can explore how relevant symbolic resources are cleverly employed to construct specific “discursive scripts” (34). To be specific, by examining narrative genres, characters, scenes, and settings of the video, the discursive script can elucidate the recontextualization of relevant social practices behind the text, enabling criticism of social practices and ideologies (35).

**Figure 1:**
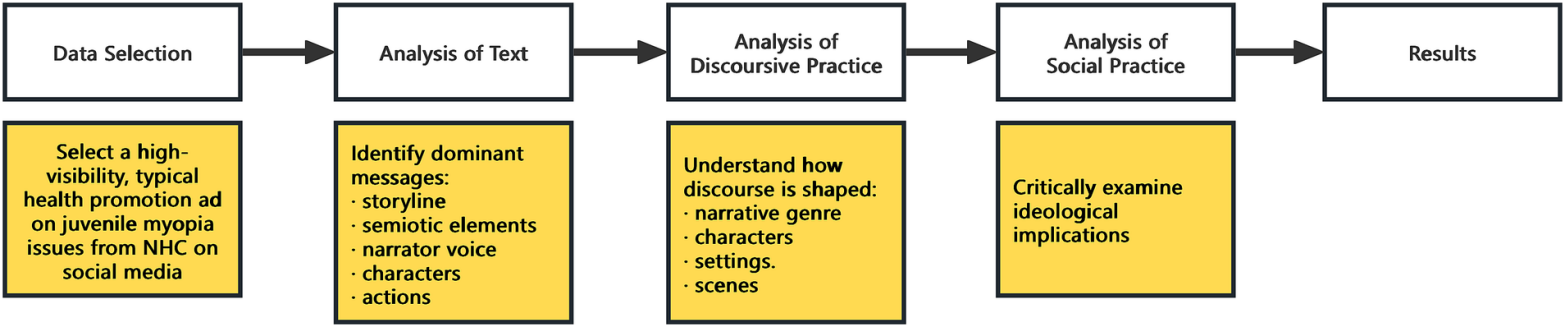
Analytical procedure of the multimodal critical discourse analysis.

The following flowchart (Figure 1) illustrates how MCDA was conducted and applied in this study. First, in terms of data selection, this research aimed to identify a representative and widely viewed public health promotion advertisement related to juvenile myopia, recently released by the National Health Commission (NHC) on social media. After a careful screening process, the selected advertisement was a public health promotion video posted by the NHC on one of China’s most popular social media and video-sharing platforms, bilibili. The advertisement was released under the account “Official Account for Healthy China,” with the title “[Public Service Advertisement] This public service advertisement is eye-catching!.” The NHC and the Ministry of Education provided joint guidance for this advertisement. At the same time, it was co-planned by the Popular Science Branch of the Chinese Medical Association, the Popular Science Branch of the Chinese Medical Doctor Association, and the China Medical We-media Association, and produced by YOULAI. This company provides medical services through the Internet. The video has been played 15 thousand times, which makes it one of the most-watched public health promotion advertisements with a “Myopia” tag posted by the NHC on bilibili. As the request for the permission to use the advertisement was not granted by the copyright holder, the advertisement images cannot be directly included in the following analysis and discussion. Readers are kindly advised to view the advertisement via the link (39) to facilitate understanding of the subsequent analysis.

After determining the data, MCDA was employed to analyze the selected advertisement. The analysis was divided into three stages, focusing, respectively, on the text, discursive practice, and social practice of the advertisement. In the analysis of the text, the researcher will concentrate on the overall content and structure of the advertisement—what kind of story it tells, what narrator voice it adopts, what characters are involved, what settings in which these characters appear, and what actions they perform. Identifying and summarizing these elements provides crucial entry points for the subsequent analysis of discursive practice. In the analysis of the discursive practice, the researcher will conduct a deeper examination of the text from four key perspectives: narrative genres, characters, scenes, and settings. This stage aims to reveal the specific strategies and techniques employed by the advertisement producers to construct meaning and deliver the intended message. Finally, in the social practice analysis, building on the findings of the previous two stages, the researcher can explore the underlying viewpoints embedded in the advertisement’s discourse, as well as their contradictions with broader social realities. This stage ultimately uncovers the ideological stance of the advertisement and its producers.

## 5 Results and discussions

This section will present the analysis of the data based on the multimodal critical discourse analysis approach with the concept of discursive script, but following the steps of Fairclough’s framework. Firstly, the text of the advertisement will be illustrated to illustrate the main content of the advertisement. Secondly, the discursive practice of the advertisement video will be analyzed, focusing on narrative genres, scenes, settings, and characters that can show how actual, concrete participants, processes and settings in the practice of preventing juvenile myopia are ideologically recontextualized or transformed in the discursive script of this advertisement; Lastly, based on these analyses, social practices and ideologies hidden in this process will be pointed out and criticized.

### 5.1 Text: what is showcased

The video features four families, each composed of an adult and a minor, presented sequentially in a parallel manner. The video begins by showcasing scenarios of four families, respectively, where adults and minors do certain activities together, namely reading, playing video games, using smartphones, and painting. Subsequently, the adult figures in each scenario become aware of their children’s unhealthy eye habits. Ultimately, they address these issues through interactive engagement with their children, behaving as models to help them steer clear of the risks of myopia. The video concludes with scenes of the four children smiling at the camera.

Throughout the video, a middle-aged male narrator provides a voiceover, using a standard Mandarin tone reminiscent of a news anchor, elaborating on the extended meaning of the video. This narrator can be seen as embodying a government spokesperson-like image. The central message conveyed by the video is that parents should act as role models, guiding their children to cultivate proper eye habits and preventing myopia in children and teenagers.

### 5.2 Discursive practice: how to recontextualize

#### 5.2.1 Narrative genres

The video adopts a genre of entertaining narrative, which can convey personal experiences on how to cope with life (36). The video with an entertaining narrative is usually structured with four stages: orientation, complication, resolution, and coda. Taking into account the parallel relationships and similar patterns in narratives among the four families in the advertisement, the following section will use one of the families presented in the video whose first appearance occurs at the 00:09 mark of the advertisement as an example, which aims to facilitate the reader’s understanding of the four stages of this entertaining narrative and the relationships between the images, text, and voiceover in the video.

In the stage of orientation, at the 00:09 mark of the advertisement, a scene from everyday life is depicted in the video: a mother and daughter sit at a dining table, engaging in design and drawing activities, respectively. It is worth noting that both the mother and daughter are too close to the laptop screen and desktop, which is considered an unhealthy eye habit. In the stage of complication, from the 00:20 to 00:24 of the advertisement, the mother notices this and expresses concern about the daughter’s incorrect eye habits. In the resolution stage, from 00:45 to 00:48 of the advertisement, the mother proactively reminds the daughter and provides a demonstration, and the child willingly accepts the mother’s reminder. In the stage of coda, at the 00:53 mark of the advertisement, the daughter is gazing at the audience with a happy smile, appealing to the audience to protect children and adolescents’ eyes from the threat of myopia. Similar processes of preventing juvenile myopia unfold in the other three families presented in the video, following the same narrative structure. Accompanying these visuals is voiceover narration explaining and extrapolating these four family scenarios, highlighting the common motivation behind the characters’ actions: correcting children’s unhealthy eye habits and assisting in preventing juvenile myopia.

In this narrative, the advertisement producers attempt to tell the audience a story: by practicing correct lifestyle and eye habits in their daily lives, parents can set a model and prevent myopia among minors. Through this video, the advertisement producers integrate this viewpoint into the audience’s everyday life, guiding them to pay attention to such a lifestyle. This narrative, which views parents’ lifestyles and modeling role as a panacea, inherently reflects a strong consumerist ideology and can be seen as the influence of neoliberalism on related discourse. In essence, it tries to persuade adult parents to be the primary individuals responsible for the prevention of juvenile myopia.

#### 5.2.2 Characters

The advertisement involves three categories of participants in the social practice of preventing juvenile myopia: adult individuals with family and children, underage individuals, and governmental authorities. They are, respectively, represented by the characters, the adults, the children, and the voiceover in the video.

In the advertisement, the adult roles, such as fathers, mothers, and elder siblings in the four featured families of the video, embody the roles of parents in real family life. In the video, these individuals engage in activities such as reading, playing video games, and using smartphones with their children, all displaying unhealthy eye habits. In this context, the discursive script revolves around adults, which contains a presupposition that parents and children can often participate in activities together and leads to a presupposed result: parents’ bad eye habits can be imitated by children, leading to myopia in children and teenagers. This shifts the primary responsibility for the severe public health issue of juvenile myopia to the parents depicted in the video and the many adults in society who have families and children, as represented in the video.

In the advertisement, the children representing real-life underage individuals are presupposed to the roles of innocence, ignorance, and obedience: they imitate the behaviors of nearby adults without hesitation and happily accept guidance and requests from their parents. The discursive script here, to some extent, denies the agency of underage individuals, emphasizing one-sidedly the imitative nature of children. This echoes the parental roles in the video, highlighting the responsibility of adult parents in preventing underage myopia.

As the potential character in the advertisement, the voiceover narration’s role is significant. Its voice, characterized by a middle-aged male announcer, represents the advertising producers and relevant governmental authority. The four sentences of the narration utilize identifying and relational processes from the systematic functional grammar’s transitivity (37), aligning with the visuals to further emphasize children’s lack of agency and parents’ absolute influence on them. The latter two sentences employ material processes, using imperative sentences that omit the actor (parents, adults) and the scope (family environment) to highlight the beneficiary (children and adolescents) and the goal (to prevent myopia). Thus, the narration creates a presupposition that preventing underage myopia becomes an inherent obligation for parents, which will not change with variations in environmental conditions. And this obligation is legitimized by the grammatical features and style of the voiceover, as well as the content of the video.

Through the above analysis based on the advertisement, it can be observed that, in the process of recontextualizing the participants involved in the social practice of preventing juvenile myopia, the advertising producers, via the images and actions of characters in the video, establish three presuppositions, which are that parents should accompany their children and be their model, children lack agency. It is only natural for parents to take on the responsibility of preventing myopia in children and adolescents. This one-sidedly highlights the role of adult individuals in this process, identifying them as the primary responsible parties for the issue. To achieve these three presuppositions and legitimize them, the advertisement producers effectively adopt a strategy of deletion (34), refusing to portray underage individuals and other relevant social actors as participants in the advertisement. Yet, both in policy texts and public health practices, these neglected actors who are not represented in the discursive script of the video should have been expected to play significant roles.

#### 5.2.3 Scences

As mentioned earlier, the advertisement comprises four stages: orientation, complication, resolution, and coda. In the first two stages, the advertisement producers primarily highlight the unhealthy eye habits depicted in the advertisement. In relevant policy texts, the NHC lists a series of unhealthy eye habits that may lead to adolescent myopia. The advertisement, through close shots displayed in the advertisement from 00:11 to 00:27, showcases the unhealthy eye habits of the children in the four families, along with the concerned expressions of the parents, to achieve the recontextualization of the social practice.

The unhealthy eye habits reflected in the four selected families in the advertisement include reading while lying down, prolonged video game sessions, using smartphones in dim lighting conditions, and having eyes too close to the desk/screen. These scenes have certain conditional limitations, typically occurring within the family setting. Advertising producers utilize these relatively specific unhealthy eye habits to achieve a substitution strategy (34). Many unhealthy eye habits, which are substituted in the video, are often associated not only with the family but also with factors such as school, society, culture, and many others.

In the stage of resolution, the advertisement producers address the four unhealthy eye habits depicted in the video by having the four adults demonstrate corrective actions for their children, who all accept them willingly. As shown in the advertisement from 00:40 to 00:50, through close shots of parents and children happily interacting in this stage, the advertisement attempts to portray this correction process as easy, smooth, and enjoyable. However, in actual social practice, interactions between children and parents are often not smooth sailing and may require parents to have sufficient family time, educational wisdom, and the child’s active cooperation, which may not be present in all families. In fact, the advertisement once again employs substitution, generalizing relatively specific situations, thus shaping an idealized discursive script.

At the end of the advertisement, the advertisement producers have the four children, who have corrected their unhealthy eye habits under parental guidance, gaze into the camera with smiles, and the video ends with a happy ending. Through close shots of this gazing and smiling pose in the advertisement from 00:51 to 00:55, the advertisement producers create a demand image (38), placing the audience and performers at a close social distance, prompting the audience to acknowledge the reality of the content in the video and looking forward to audience’s responses and social interactions, especially expecting responses from adults with families in real life. This design effectively emphasizes once again the individual responsibility of adults in preventing myopia in juvenile individuals.

Based on the above description, in the discursive script of the advertisement, advertisement producers utilize specific processes to achieve substitution for real-life situations and incorporate close shots in specific processes to accomplish the recontextualization of social practice. This highlights the responsibility of adults within the family and encourages them to play a crucial role in preventing myopia in underage individuals.

#### 5.2.4 Settings

In the advertisement, it is also noticeable that all characters appearing in the advertisement, are set in family settings. These environments often feature spacious and bright living rooms, large screens, and video game consoles, as well as carefully decorated walls and lofts dedicated to activities, among other elements. These settings imply that the four families depicted in the advertisement have living conditions and environments above the average for urban residents in China. The settings reflect the socioeconomic status of these families, which may be closer to the middle class or even higher in major Chinese cities. Such socioeconomic status typically provides better conditions for their children in terms of healthcare, including prevention and treatment of myopia.

The advertisement creators utilize settings to create representations of middle-class families in major Chinese cities, using these specific families to substitute more common ones. This recontextualization, to some extent, presents a model to the audience, implying to viewers, especially adults with families, that they should strive to emulate the families in the video and take proactive responsibility for preventing myopia in underage individuals. Moreover, considering that the four families featured in the advertisement are all from middle-class backgrounds in large cities, this design appears to be attempting to conceal the socioeconomic differences between different families, such as parental occupation, income, education level, as well as differences in community environment, living conditions, and their impact on adolescent myopia.

### 5.3 Social practices: what ideas are reflected

Through the above analysis, it can be seen that the advertisement creators utilize techniques such as deletion and substitution to design narrative genres, characters, scenes, and settings, successfully recontextualizing the prevention of myopia in underage individuals. This recontextualization of the social practice of preventing myopia in underage individuals transforms the discourse of this advertisement into a form of biopedagogical discourse characterized by neoliberalism.

In the video, although there are adult roles and child roles within the family context, as well as the potential role of voiceover, the entire discursive script is designed to emphasize the important responsibility of adults in preventing myopia in minors. Child roles in the video are portrayed as innocent, lacking agency, and eager to receive guidance and protection. They are positioned as “victims” of imitating parents’ unhealthy eye habits, further highlighting the crucial role of adult figures in setting examples. The voiceover employs specific speaking styles and grammar features, closely coordinating with the video content, to further rationalize the demands placed on adult roles and adult viewers. Essentially, these contents demand that adults in society take primary responsibility for preventing myopia in minors as a public health issue. Meanwhile, other stakeholders, such as minors themselves, schools, healthcare institutions, and society, which are emphasized in related policy documents like The Comprehensive Implementation Plan for the Prevention and Control of Myopia in Children and Adolescents (6) and The Action Plan for the Prevention and Control of Myopia in Children and Adolescents 2021–2025 (18), are not represented in the video. The discourse of the advertisement solely interacts with the policy discourse emphasizing adult parents’ personal responsibility. This one-sided intertextuality actually implies that in the process of promoting myopia prevention among minors, information is selectively provided to the public, while the complexity of the issue is obscured, thereby reinforcing the idea that the primary responsibility for social welfare lies within the family unit (24).

The advertising discourse in the video also utilizes various symbolic resources to construct an idealized family from the discursive script, portraying parents as intelligent educators who effortlessly ensure their children happily accept their guidance in a spacious, bright, digitally equipped, and exquisitely decorated home, reflecting the middle-class lifestyle typical of major cities. However, such a portrayal clearly deviates from reality. While it is true that the proportion of myopia in urban areas is higher in China, not all urban residents can enjoy similar living conditions depicted in the advertisement. Most urban and rural residents lack the excellent hardware facilities showcased in the video and cannot guarantee sufficient time to accompany their children or provide them with proper knowledge on myopia prevention and suitable family education guidance. When the families depicted in the advertisement are presented as role models, it actually reflects the absence of the discourse power of the middle and lower classes, especially the ordinary urban working class, in related discourses.

The discursive script characterized by the aforementioned features and connotations not only achieves a recontextualization of the issue of preventing myopia in minors in advertising but also embodies a framework for evaluating relevant social actions (34). In the public health promotion advertisements discussed in this paper, a biopedagogical discourse revolving around myopia in minors is reconstructed through “selectively appropriate, relocate, refocus and relate to other discourses to constitute its own order and orderings” (34), which reflects a neoliberal standpoint. This standpoint demands that adult citizens with families in society take responsibility while downplaying the complexity of public health issues and neglecting the diversity of different individuals across different social dimensions. This tendency can exacerbate the unfairness towards marginalized groups in society.

## 6 Conclusion

This study employs a multimodal critical discourse analysis (MCDA) within Fairclough’s three-dimensional framework to examine a public health promotion advertisement about juvenile myopia issues released by China’s National Health Commission (NHC) on Bilibili. Through an analysis of narrative genres, characters, scenes, and settings, the study revealed how the advertisement constructs a biopedagogical discourse aligned with neoliberal ideology—promoting individual responsibility while downplaying broader social and institutional roles in myopia prevention.

The findings show that the advertisement recontextualizes middle-class urban family life as the normative ideal, marginalizing working-class and rural experiences. It emphasizes parental responsibility through carefully curated visuals and narrative framing, while omitting structural factors such as socioeconomic status, educational disparities, and institutional accountability.

This study contributes to understanding how public health messages are not neutral but ideologically charged. It demonstrates how such discourse subtly encourages self-governance and compliance through emotionally resonant, yet socially selective, representations. These insights are valuable not only for scholars of discourse and ideology but also for policymakers, pediatric ophthalmologists, and health communication professionals. Understanding these discursive mechanisms can help them critically evaluate and improve the design of future health campaigns, ensuring that messages are inclusive, socially aware, and responsive to the diverse realities of target populations.

This research is limited by its focus on a single advertisement. While the selected video can be representative and widely circulated, enriching the study of public health discourse in non-Western contexts, it does not fully reflect the diversity of public health discourses across different platforms, regions, or institutional sources. Future studies should expand the dataset to include multiple advertisements, platforms, and potentially cross-national comparisons to better understand the ideological dynamics at play.

## Data Availability

The original contributions presented in the study are included in the article, further inquiries can be directed to the corresponding author.
